# Home visiting and perinatal smoking: a mixed-methods exploration of cessation and harm reduction strategies

**DOI:** 10.1186/s12889-016-3464-4

**Published:** 2016-08-11

**Authors:** Heather Griffis, Meredith Matone, Katherine Kellom, Erica Concors, William Quarshie, Benjamin French, David Rubin, Peter F. Cronholm

**Affiliations:** 1 PolicyLab, The Children’s Hospital of Philadelphia, 3535 Market, Suite 1424, Philadelphia, PA 19104 USA; 2 Division of General Pediatrics, The Children’s Hospital of Philadelphia, Philadelphia, PA USA; 3 Department of Biostatistics and Epidemiology, University of Pennsylvania, Philadelphia, PA USA; 4 Department of Pediatrics, University of Pennsylvania Perelman School of Medicine, Philadelphia, PA USA; 5 Department of Family Medicine and Community Health, University of Pennsylvania Perelman School of Medicine, Philadelphia, PA USA

**Keywords:** Smoking cessation and reduction, Home visiting, Maternal and child health

## Abstract

**Background:**

Home visiting programs represent an important primary prevention strategy for adverse prenatal health behaviors; the various ways in which home visiting programs impact prenatal smoking cessation and reduction behaviors remain understudied.

**Methods:**

Mixed methods approach using a retrospective cohort of propensity score matched home visiting clients and local-area comparison women with first births between 2008–2014 in a large Northeast state. Multivariable logistic and linear regression estimated third trimester prenatal tobacco smoking cessation and reduction. Additionally, qualitative interviews were conducted with 76 home visiting clients.

**Results:**

A program effect was seen for smoking cessation such that clients who smoked less than ten cigarettes per day and those who smoked 20 or more cigarettes per day during the first trimester were more likely to achieve third trimester cessation than comparison women (*p* <0.01 and *p* = 0.01, respectively). Only for heavy smokers (20 or more cigarettes during the first trimester) was there a significant reduction in number of cigarettes smoked by the third trimester versus comparison women (*p* = 0.01). Clients expressed the difficulty of cessation, but addressed several harm-reduction strategies including reducing smoking in the house and wearing a smoking jacket. Clients also described smoking education that empowered them to ask others to not smoke or adopt other harm reducing behaviors when around their children.

**Conclusions:**

While a significant impact on smoking cessation was seen, this study finds a less-clear impact on smoking reduction among women in home visiting programs. As home visiting programs continue to expand, it will be important to best identify effective ways to support tobacco-related harm reduction within vulnerable families.

**Electronic supplementary material:**

The online version of this article (doi:10.1186/s12889-016-3464-4) contains supplementary material, which is available to authorized users.

## Background

Prenatal smoking is a modifiable at-risk behavior and a major target of public health agencies and evidence-based interventions across the US. Health effects of smoking during pregnancy include increased likelihood of low birth-weight babies, pre-term delivery, infant mortality, and sudden infant death syndrome [1–3]. Evidence-based prenatal smoking cessation programs are an important public health intervention for minimizing the harmful effects of prenatal smoking on mothers and infants. Successful interventions vary in approach and include the American College of Obstetricians and Gynecologists’ (ACOG) 5 A’s prenatal smoking cessation approach [4, 5] and the Smoking Cessation and Reduction in Pregnancy Treatment Method (SCRIPT) [6]. The 5 A’s and SCRIPT are clinic-based programs that can be integrated into prenatal office visits and offer smoking assessments and counseling to encourage smoking cessation.

Home visiting programs are uniquely positioned to reduce prenatal smoking. The federal Maternal, Infant, and Early Childhood Home Visiting Program (MIECHV) reached approximately 115,500 parents and children in 787 counties across all 50 States, the District of Columbia, and five US territories in 2014 [7]. The evidence-based programs selected for funding through MIECHV have demonstrated positive effects on a range of maternal health outcomes, including prenatal smoking cessation, pregnancy-induced hypertension and shorter inter-birth intervals. These programs also have a strong evidence base for child well-being outcomes, including improved health care utilization and school readiness [8]. Home visiting programs offer various smoking cessation intervention strategies, including client education of smoking harms and cessation strategies, motivational interviewing, and referral to outside programs that offer smoking cessation counseling. While researchers have demonstrated increased rates of smoking cessation among participants of home visiting programs, research exploring other ways home visiting programs impact prenatal smoking behaviors is lacking.

Specifically, it is uncertain whether program effectiveness differs by client baseline smoking status, including the level and history of tobacco use at program entry, or whether and how specific intervention strategies are implemented and responsive to client context (for example, the use of harm reduction versus cessation strategies for baseline heavy tobacco users). Harm reduction, reducing harm through adjustments in environmental tobacco smoke exposures, is a useful intervention approach in consideration of the challenges to full and continued cessation and the possible presence of other smokers in the home [9, 10]. The use and impact of harm reduction within home visiting has not been well documented.

This study provides an in-depth analysis of the intersection between home visitation and smoking behaviors using a mixed-methods approach with data from the Pennsylvania MIECHV Evaluation. We explore the program effect of home visiting on smoking cessation and reduction between the first and third trimester of pregnancy using quantitative data from the Nurse Family Partnership (NFP) home visiting program. We augment the quantitative analyses with a qualitative evaluation of interview data from clients across four evidence-based home visiting programs (NFP), Parents as Teachers (PAT), Healthy Families America (HFA), and Early Head Start (EHS) to investigate client perspectives on the impact of program curriculum on behavior.

## Methods

This study used a mixed methods design to explore the mechanisms and drivers for positive behavior change [11, 12] within maternal and child home visiting programs. To place this work in the growing field of mixed methods research, our study represents a partially mixed concurrent equal status design, in which the qualitative and quantitative data were analyzed separately and mixed at the stage of interpretation [13]. By utilizing complementary quantitative and qualitative data, we are able to triangulate results to explore areas of convergence and divergence [14], building a more comprehensive understanding of program effects. Mixing methods allowed for significance enhancement [15], enabling us to extended analyses to family and social environmental factors that may explain quantitative program impacts on smoking.

### Study sample

As part of a state-wide, mixed method evaluation, we analyzed quantitative client data from 22 NFP home visiting programs across the Commonwealth of Pennsylvania, as well as individual, semi-structured interviews with 76 clients from a representative sample of four home visiting programs (NFP, PAT, HFA, and EHS; 11 sites total). Enrollment timing in each of the four programs varies—from prenatally for NFP to up to 3 years of child–age for EHS. Duration of the program also varies—NFP clients receive services for up to 2.5 years; HFA provides services to families up to when the child turns 5 years of age. Likewise, smoking cessation and reduction curricula varies and includes techniques ranging from brochures and educational materials to motivational interviewing to referral to outside smoking cessation programs.

The primary sources of quantitative data were: enrollment history of clients participating in 22 NFP programs throughout Pennsylvania, birth certificate files from the Pennsylvania Department of Public Health, welfare eligibility files from the Department of Public Welfare, and interview data.

NFP clients who delivered first-born infants between January 1, 2008 and December 31, 2014 were the target population of treatment-exposed mothers. Non-clients delivering a first-born infant between January 1, 2008 and December 31, 2014 and received welfare assistance from the Commonwealth of Pennsylvania within 12 months prior to the first infant’s birth were eligible to serve as comparison mothers. Clients and comparison women were linked to birth certificate and welfare files using an iterative deterministic linkage process that linked home visiting enrollment data to birth certificate and welfare eligibility data.

Clients were matched to comparison women using a previously described propensity score matching approach [16]. Multivariable logistic regression models estimated the expected probability of program participation for each woman within each of the 22 program catchment areas using maternal sociodemographic characteristics. Variables in the propensity score model include: maternal age at birth, race/ethnicity, education, marital status, TANF and/or foodstamp receipt prior and/or during the first trimester of pregnancy, and history of gestational diabetes or hypertension. The result was an expected probability (or propensity score) of program participation for each woman in an agency catchment area.

Matching was performed using the MatchIt program within the R software package, Version 3.2.1. Any nearest neighbor within a caliper of 0.05 was considered a match (up to a maximum of four comparison women per client). Matching was forced on catchment area, infant year of birth, and maternal age (less than 18 years of age at birth or 18 and older).

Characteristics for clients and comparison women in each agency were examined for balance across all covariates after matching [17]. As a threat to residual bias, imbalances in point estimates were identified using standardized mean differences between clients and comparison women with a threshold of 2.5 absolute percentage points and minimized for each agency through adjustment of the propensity score models. Adjustments included interaction terms between a balanced and imbalanced variable to improve balance. From this cohort, clients and comparison women were eligible for analysis if they reported smoking during the first trimester of pregnancy on the birth certificate.

To recruit for the qualitative study component, we purposively sampled from the MIECHV PA funded sites based on location, urbanicity, and program type to ensure the data represented a variety of client experiences. We recruited clients via phone and e-mail with the help of program staff, who provided contact information. To aide with recruitment, a flyer describing the study, what participation involved, and study staff contact information was provided to sites. Any client currently receiving services from one of the 11 programs who spoke English or Spanish was eligible to participate. We maximized our simple sampling scheme by interviewing as many clients from each selected site that expressed interest and scheduled an interview [18]. All clients were verbally consented prior to the interview. The semi-structured phone interviews were conducted by two qualitative research staff at the participant’s convenience, lasted approximately 1 hour, and covered how the client learned about and joined the program (initiation of services), the relationship with their home visitor and the program (engagement), their involvement with other services and review of four specific outcomes, including smoking cessation. Each participant received a $20 gift card as compensation.

Standard approaches to qualitative methods use the concept of saturation to determine sample size, which can vary greatly based on the study’s qualitative approach [19], scope, homogeneity within the sample, and data quality [20]. Our study team assessed saturation in the data when no new themes were identified as final interviews were conducted and coded at the broad code level. While smoking cessation was an area discussed in interviews, the smoking behavior of participants did not drive sampling or our assessment of saturation. Our current, post-hoc analysis of subgroups (cessators, non-cessators, and non-smokers) does meet the minimum sample size recommendation for interviewing (≥12 participants) and nested sampling design (≥3 participants) [18].

### Outcome measures

The quantitative analysis included two primary outcome measures ascertained from birth certificate fields: 1) change in number of cigarettes smoked in the third trimester compared to the first trimester; and 2) smoking cessation defined as zero reported cigarettes smoked in the third trimester followed reported use of cigarettes (*n* >0) in the first trimester.

The qualitative study component aimed to explore client perspectives on the impact of services and areas where programming could be improved. The interview covered how the program addressed smoking behaviors and whether the approach influenced behaviors.

### Analysis

The primary exposure was NFP program participation (yes/no). Multivariable linear (change in smoking behavior) and logistic (smoking cessation) regression models examined the association between primary outcomes and program participation. Mothers age at child’s birth (<18), marital status, and prior Temporary Assistance for Needy Families (TANF) receipt were included as adjustment covariates due to imbalance created by subsampling prenatal smokers from the larger propensity score matched cohort. Baseline smoking status was coded as 1–9 (light smokers), 10–19 (moderate smokers), or 20 or more cigarettes smoked in the first trimester (heavy smokers). An interaction term between NFP program participation and baseline smoking status was included to explore the difference in reduction and cessation between clients and comparison women in relation to baseline smoking status. Results were expressed as marginal probabilities of smoking cessation and marginally standardized change in cigarettes smoked [21]. Analyses were conducted using Stata version 13.0.

All interviews were coded using NVivo 10. We began coding client interviews after data collection was complete. To develop our codebook, our multi-disciplinary team met to review our individual open coding on a representative set of interview transcripts to develop a set of emerging themes and definitions. This process was repeated when we started coding the client interviews. Code definitions were adjusted for client data and new codes were added to the codebook as the themes evolved and emerged in this new data. Smoking cessation was a pre-determined quantitative outcome of the PA MIECHV evaluation. As such, a priori codes related to smoking were used and refined following review of qualitative data. We utilized a constant comparative approach involving an iterative review of primary data and coding structures and definitions resulting in refinement and finalization of the final coding schema. Members of both the quantitative and qualitative portions of the study team reviewed data related to smoking among home visiting clients. Elements of the quantitative approach informed the qualitative coding in terms of identifying factors driving analyses based on attributes such as clients being identified as smokers, reducers, and non-smokers based on their behaviors during pregnancy. This process elucidated the value of adding/describing the perspective of non-smoking clients on the relevance/importance of smoking curriculum, as well as the barriers faced by clients related to quitting. Over the course of coding the team met on a regular basis to discuss the application and definition of the coding schema. Twenty percent of the data were coded by multiple coders using inter-rater agreement to identify and correct areas of disagreement to assure coding reliability and accuracy. Discrepancies in the application of codes were discussed and resolved through group consensus.

Interview content related to smoking behaviors and related curricular impact on this outcome were collected in a single node. Themes identified through a constant comparative approach were used to help contextualize the quantitative work in the client experience and augment understanding of programmatic impact [22].

Approval for the study was granted by Pennsylvania’s Department of Public Welfare. Ethical approval was granted by the Children’s Hospital of Philadelphia’s Institutional Review Board.

## Results

### Cohort characteristics

From 2008 to 2014, 8986 clients were enrolled in 22 NFP program agencies. Clients with missing values on any covariates were dropped (*n* = 25). Acceptable comparison matches were not found for 20 clients; these clients were excluded from the analysis. A total of 2595 clients met inclusion criteria of smoking in the first trimester. The final sample contained 10,296 women: 2595 clients matched to 7701 comparison women.

Compared to the total cohort of clients and comparison women, the smoking cohort was more likely to be of white race, younger at the time of first birth, and have less than a high school education (Table 1). Because clients were matched to comparison women based on smoking status prior to the start of pregnancy, there was imbalance in mothers’ age at child’s birth (<18), marital status, and prior TANF receipt. These factors were included in the multivariate models to account for this imbalance.

**Table 1:** Characteristics of NFP clients and comparison women, 2008-2014

	Smoking cohort			All clients/comparisons		
Characteristics	Comparisons (*n* = 7701)	Clients (*n* = 2595)	*p-*value	Controls (*n* = 33,384)	Clients (*n* = 8986)	*p-*value
	%	%		%	%	
Age, less than 18 years	28.9	24.2	<0.01	18.6	22.1	<0.01
Race/ethnicity						
White	75.3	75.2		50.9	50.3	
Black	12.9	13.8		25.3	25.9	
Hispanic	10.5	10.2		20.9	21.3	
Other	1.4	0.8	0.08	2.9	2.6	0.22
Unmarried	93.8	92.5	0.03	89.8	88.9	0.01
Education, less than high school	36.7	38.2	0.19	30.5	31.2	0.20
TANF Receipt	57.1	54.0	0.01	49.6	50.4	0.18
Foodstamp Receipt	59.8	60.1	0.76	48.9	47.2	<0.01

Table 2 compares women who stopped smoking during the third trimester and women who did not. Women who cessated tended to be of black race (*p* <0.01), married (*p* <0.01), have less than a high school education (*p* <0.01), and less likely to receive foodstamps prior to birth (*p* <0.01).

**Table 2:** Characteristics of smoking cessators and non-cessators in third trimester, 2008-2014

Characteristics	Cessators (*n* = 2630)	Non-cessators (*n* = 7432)	*p-*value
Age, less than 18 years	26.4	24.6	0.08
Race/ethnicity			
White	72.8	77.7	
Black	14.2	11.9	
Hispanic	11.4	9.3	
Other	1.6	1.1	<0.01
Unmarried	94.9	93.2	<0.01
Education, less than high school	32.6	38.7	<0.01
TANF Receipt	55.2	57.1	0.10
Foodstamp Receipt	54.4	61.8	<0.01

Seventy-six home visiting clients were interviewed as part of the qualitative study. Fifty-five (72 %) participants reported that they did not smoke, 19 (25 %) reported that they did smoke, and 2 (3 %) did not report. Of the participants who smoked at the start of the program, 11 reported not changing their smoking patterns. Four participants reported achieving cessation and four described reducing the number of cigarettes at some time during the program. As this paper aims to discuss the impact of home visiting smoking curriculum on clients and families, we analyzed and include data from smokers and non-smokers. Demographic characteristics of all interviewees are provided in the Additional file 1. Clients who smoked were more likely to be white, over the age of 18, and have less than a college degree. In the quotes that follow, we provide the participant’s ethnicity and age. Additional examples from our interview data appear in the Additional file 1. Bolded text signifies that it also appears in the text below.

### Smoking cessation

#### Quantitative

A program effect was seen for smoking cessation among light (less than ten cigarettes during the first trimester) and heavy (20 or more cigarettes during the first trimester) baseline smoking clients. Clients who were light baseline smokers had a 45 % probability of smoking cessation compared to 38 % for comparison women (*p* <0.01; Table 3). Heavier baseline smoking clients also had a higher probability of smoking cessation—16 % compared to 12 % (*p* = 0.01).

**Table 3:** Marginal probabilities of smoking cessation and marginal change in number of cigarettes smoked between first and third trimester^a^

	Clients	Comparisons	
	Estimate (95 % CI)	Estimate (95 % CI)	*p-*value
Probability of smoking cessation			
<10 cigarettes per day at baseline	0.45 (0.42, 0.49)	0.38 (0.37, 0.40)	<0.01
10–19 cigarettes per day at baseline	0.22 (0.19, 0.25)	0.20 (0.19, 0.22)	0.43
≥20 cigarettes per day at baseline	0.16 (0.13, 0.19)	0.12 (0.11, 0.13)	0.01
Change in number of cigarettes smoked			
<10 cigarettes per day at baseline	−2.0 (−2.2, −1.8)	−1.8 (−2.0, −1.8)	0.17
10–19 cigarettes per day at baseline	−5.0 (−5.4, −4.7)	−4.8 (−5.0, −4.6)	0.23
≥20 cigarettes per day at baseline	−14.0 (−14.9, −13.1)	−12.5 (−13.0, −12.0)	<0.01

^a^Estimate obtained from multivariable regression model, adjusted for maternal age at birth, marital status, and prior receipt of Temporary Assistance for Needy Families (TANF)

Qualitative: Four of the 19 clients who smoked described achieving cessation, with three suggesting that their behavior change was motivated primarily by their pregnancy rather than a direct program effect.

*They actually help – well, kind of gave me suggestions of how to quit smoking with all my pregnancies. But I mainly did that by myself.* (White, 21)

*I don’t smoke anymore. … [My home visitor] helped me realize how really – how big of a risk it was to him… I knew not to smoke around him and not to while I was pregnant. I knew that was harmful. But I didn’t realize that it can even get into – I guess like, into the walls and things like that, the room that he’s in. I didn’t realize that was important, also.* (Black, 22)

Like the other cessators, this client described understanding the negative health impacts of smoking during pregnancy, but attributed her success with cessation to the knowledge she gained from the program about third-hand smoke (THS - i.e. residual nicotine and toxins from smoked cigarettes left on surfaces and fabrics).

### Smoking reduction and behavior change

#### Quantitative

While a program effect was not seen for light or moderate baseline smokers, clients who were heavy smokers significantly reduced the number of cigarettes smoked during the third trimester—clients reduced the number of cigarettes by 14, while comparison women reduced by 12.5 cigarettes (*p* <0.01; Table 3).

#### Qualitative

Clients who smoked described the different ways home visitors supported reduction, from answering client questions to providing contacts for cessation support.

*I did cut back tremendously when I was pregnant – per [home visitor’s] advice, and also, quit smoking in the house entirely and the car. …[W]e talked a lot about SIDS [Sudden Infant Death Syndrome] just because I was so inquisitive about it because I was a smoker. Anything negative that I wanted to learn and about the effects and what the best way was to quit, and hotlines I could call, and discuss with my doctor, and steps I should take to quit.* (White, 33)

Maintaining this behavior change, however, was described as a “constant struggle” and, as the client above continues:

*[A]fter I was able to smoke again I went to town.*

Of the eight interview participants who described cessating (*N* = 4) or reducing (*N* = 4), only two described successfully maintaining their behavior change.

The impact of educating families about other behavioral changes aimed at reducing harm to children emerged as a major theme in the qualitative interviews. Over half (53 %; 10 of 19 clients who smoked) and 29 % (16 of 55 non-smoking clients) mentioned reducing the harm of smoking. Clients described learning about the risks of and ways to avoid exposing children to second- and THS. Program-associated exposure to this information was described as helping many clients decide to stop smoking in the house, in the car, or in the presence of their children. Clients mentioned adopting the practice of wearing a smoking jacket they would remove before coming in to the house, and/or immediately washing their hands before interacting with their children.

*We talked about third-hand smoke. […] she suggested the smoking jacket, which is the hoodie we keep outside for when we’re smoking. Because I have like three, four feet of hair and it’ll trap everything so – put the hoodie on when we go outside and have a cigarette and then take it off before we come in and wash our hands and everything. […] I didn’t even know third-hand smoke was a thing.* (White, 34)

*I know that secondhand smoke is bad for my child which is why I smoke outside, and I wear a jacket and that jacket is the designated smoking jacket.* (White, 20)

The program provided new, useful information that helped parents reduce harm to their children with behaviors operating outside of their cessation success.

Additionally, a number of clients (smokers and non-smokers) described how program impact empowered them to ask friends and family members to adopt harm reducing behaviors. For example, one non-smoker described how her home visitor taught her about smoke staying on clothing and, unexpectedly, found the information useful when a family acquaintance who smokes asked to hold her son.

*I didn’t really expect to be around anybody that smoked. But, with the information she gave me, I felt comfortable saying that I wasn’t comfortable with that person holding him.* (White, 26, non-smoker)

Similarly, non-smokers with family members who smoke described how this information impacted the behaviors of husbands/partners and grandparents to reduce the exposure of children to second and THS. Other non-smokers suggested that the knowledge they gained made them avoid public spaces where their children might be exposed.

### Barriers to change

Qualitative data provided us the opportunity to explore what made smoking curriculum less effective for clients who were unable to cessate, reduce, or maintain their behavior change. Many smokers described knowing and understanding the potential harms of smoking, but felt dependent on cigarettes for stress relief.

*[T]o be honest, a cigarette is like a relief to me. Like when I’m so stressed out I light a cigarette up, I smoke it and, poof, I’m relieved from that problem. I mean, that’s just how I feel. I don’t, you know, I don’t do nothing else except for cigarettes.* (Hispanic, 26)

*The cigarette is like, hey, this is my getaway stress reliever compared to hey, I don’t got this and I used to do it. I don’t have nothing now. I can’t go outside and have that couple minutes to calm down type of thing.* (White, 21)

These clients described smoking as an important coping mechanism that provided a temporary, but much needed escape. Another barrier, which for some was related to using cigarettes to relieve stress, was the individual’s readiness to change. Very simply,

*I think some people just don’t wanna quit.* (White, 20)

And,

*people are going to do what they’re going to do. (White, 29)*

This sentiment was echoed by smokers and non-smokers. A number of clients questioned whether the program could be effective at changing smoking behaviors for this reason.

*I think a lot of people are addicted to smoking. And if they’re not – if they don’t wanna quit, somebody telling them the risks and the harmfulness is not gonna change anything. [The program] could address it, but as far as any change occurring? I don’t see that happening with the family unless, like I said, unless they are – I think the harmfulness of it should be addressed, but if the parents aren’t willing to change, it’s not gonna happen.* (White, 34, non-smoker)

Another mediator of successful behavior change clients discussed was their home visitor’s attitude and approach to addressing cessation. While some clients highlighted their home visitors’ non-judgmental approach, a few cautioned that this conversation could feel uncomfortable and hurt the relationship.

*So we have been trying to avoid those subjects. I mean, some of us in the house is willing to go outside, and it’s – there’s been multiple conversations about smoking and secondhand smoke and they really don’t feel comfortable because mainly it sounds like, “Hey, you need to quit.” And not everybody’s willing to quit.* (White, 21)

*People that smoke, I consider that an addiction. So I almost think it would be more helpful for them to link them to resources for smoking cessation rather than making them feel guilty for their second-hand smoke affecting their children. And I think that’s the affect it would have.* (White, 31, non-smoker)

This perspective suggests that programs may need to be flexible and open-minded on this issue to avoid alienating clients and family members.

## Discussion

This mixed methods exploration of smoking behaviors among home visiting clients illustrates the challenges and nuanced ways home visiting programs influence client behaviors related to smoking cessation and reduction. Overall, we found a significant program impact for prenatal smoking behaviors among program participants regarding smoking cessation; clients were more likely to achieve cessation during the third trimester versus comparison women. Consistent with prior findings [16], these results highlight the efficacy of evidence-based home visiting programs on maternal smoking cessation with qualitative support for attribution of program effect.

This analysis found a less clear impact of evidence-based home visiting programs on prenatal smoking reduction, similar to previous studies [23, 24]. Clients only experienced a significant reduction in the absolute number of cigarettes smoked between the first and third trimester versus comparison women among those who were heavy baseline smokers. This may be due to light baseline smokers’ relative ease in the ability to quit compared to heavy smokers, and thus program effects are seen in heavier smokers through reduction rather than cessation as reflected in other literature [25]. Given that reduction during pregnancy among heavy smokers has been shown to have greater positive effects on outcomes than light smokers, focusing efforts to reduce smoking among these mothers may be more feasible, rather than quitting completely [26, 27]. Additionally, providing different treatments and services based on smoking habits may result in better outcomes.

This finding was also reflected in the qualitative data--the majority of interviewed clients who smoked did not change their tobacco intake. Instead, interviews suggested effective behavior changes encouraged by the program to help clients reduce smoking exposure and associated harm to children. Additionally, information about the impact of second- and third-hand smoke is relevant to families where family and friends smoke, not just for mothers who smoke. By universally providing this information to families, home visiting programs empower parents to reduce harm to their children with sensitivity to variations in local context.

As for second-hand smoke, infants and children are at increased risk from THS through exposure to cigarette compounds and their associated deleterious effects on child health and development, including decreased lung growth and more frequent sickness [28]. The results of a systematic review of interventions designed to change smoking behaviors in families with young children suggests that programs focusing on reducing exposure to second hand smoke were more successful than those targeting smoking cessation. This work called for more investigation of the context of an entire family and social network [29]. Focusing on harm reduction and the smoking behaviors of a family unit is an important way to study the impact of home visiting programs on maternal and child health.

Children’s THS exposures are driven by children being more often indoors, on the floor, and putting non-food items in their mouths. Additional disproportionate risk factors for THS exposures include infants’ and children’s less developed respiratory and immune systems and increased respiratory rate relative to body size [28]. Qualitative results suggest that clients unable to decrease tobacco use are potentially benefitting from a harm reduction approach. Education about environmental tobacco smoke exposure can more comprehensively address the impact of smoke on child health. Additionally, program evaluation measurements may need to incorporate a wider array of benchmarks to fully understand how programs affect various health behaviors that relate to smoking and family health.

Lastly, interviews provided insight into the experiences of women who described feeling unwilling or unable to achieve cessation. Similar to prior studies, stress [30, 31] and individual readiness to change behavior impacted one’s ability to initiate and maintain cessation. Exposure to program smoking curriculum is not always enough to impact individual readiness or emotional investment in behavior change. For these individuals, it is important for home visitors to prioritize their relationship with the client. An unpleasant experience could have a long-lasting, negative impact on the client-home visitor relationship and undercut other program messaging and success.

### Limitations

There are several limitations to this study. The observational study design is subject to bias is estimates of program effect. However, the use of propensity score matching to control for measured differences between clients and the comparison group minimizes this concern. Observational study designs utilizing propensity score matching represent a valuable method for large-scale program evaluation following dissemination, as the utility and practicality of experimental evaluation designs in public health program dissemination are limited [32–34]. In addition, self-reporting on birth certificates could have limited our ability to accurately capture self-reported behaviors. Self-reported smoking on birth certificates has been found to underrepresent actual smoking behaviors. However, the estimates of smoking prevalence in this study sample might be less vulnerable to underrepresentation, because Medicaid-enrolled women, younger women, and women with less educational attainment are less likely to misreport smoking behaviors [35, 36]. Lastly, designed to assess effectiveness, our analysis did not take dosage of this or other social service programs into consideration.

## Conclusions

While a significant impact on smoking cessation was seen, this study finds a less-clear impact on smoking reduction among women in home visiting programs. Given that smoking cessation is a difficult outcome to achieve, curricula of home visiting programs may be better enhanced to include smoking reduction techniques to reduce the harm of smoke on young children. Additionally, home visiting programs may exhibit positive outcomes when collaborating with outside smoking cessation and reduction programs as an addition to the home visiting program itself. As home visiting programs continue to expand, it will be important to identify effective ways to support tobacco-related harm reduction within vulnerable families.

## Abbreviations

EHS, early head start; HFA, Healthy Families America; MIECHV, maternal, infant, and early childhood home visiting programs; NFP, nurse-family partnership; PAT, parents as teachers; TANF, temporary assistance for needy families; THS, third-hand smoke

## Additional file

Additional file 1: (DOCX 28 kb)
