# Supplementary material for: Home visiting and perinatal smoking: a mixed-methods exploration of cessation and harm reduction strategies
Source: BMC Public Health. 2016 Aug 11;16:764. doi: 10.1186/s12889-016-3464-4 (PMC4982407; doi:10.1186/s12889-016-3464-4)
Supplement: Additional file 1: — (DOCX 28 kb) [file 12889_2016_3464_MOESM1_ESM.docx]

Appendix 1. Client Interview Characteristics

|  | n | % |
| --- | --- | --- |
| Sex |  |  |
| Female | 71 | 93.42 |
| Male | 5 | 6.58 |
| Education |  |  |
| Some HS, no degree | 13 | 17.11 |
| High School | 25 | 32.89 |
| Some College, no degree | 21 | 27.63 |
| Associate's | 7 | 9.21 |
| Bachelor's | 8 | 10.53 |
| Master's | 1 | 1.32 |
| Professional | 1 | 1.32 |
| Number of Children |  |  |
| Pregnant | 4 | 5.26 |
| 1 | 45 | 59.21 |
| 2 | 15 | 19.74 |
| 3 | 8 | 10.53 |
| 4 | 1 | 1.32 |
| 5+ | 1 | 1.32 |
| Grandparent | 2 | 2.63 |
| Race |  |  |
| African | 4 | 5.26 |
| Asian | 1 | 1.32 |
| Black | 25 | 32.89 |
| White | 43 | 56.58 |
| Other | 3 | 3.95 |
| Ethnicity |  |  |
| Hispanic | 4 | 5.00 |
| Non-Hispanic | 72 | 95.00 |
| Relationship |  |  |
| Single | 39 | 51.32 |
| Married/ Partnered | 34 | 44.74 |
| Separated/ Divorced | 3 | 3.95 |
| Urbanicity | N |  |
| Urban | 39 | 51.32 |
| Rural | 37 | 48.68 |
| Age |  |  |
| 15-19 yrs | 4 | 5.26 |
| 20-24 yrs | 27 | 35.53 |
| 25-29 yrs | 22 | 28.95 |
| 30 -34 yrs | 17 | 22.37 |
| 35-39 yrs | 4 | 5.26 |
| 40 + yrs | 2 | 2.63 |
| Program |  |  |
| EHS | 11 | 14.47 |
| HFA | 11 | 14.47 |
| NFP | 36 | 47.37 |
| PAT | 18 | 23.68 |
| Language |  |  |
| English | 69 | 90.79 |
| Creole | 3 | 3.95 |
| Spanish | 1 | 1.32 |
| Other | 3 | 3.95 |
| Employment |  |  |
| No | 43 | 56.58 |
| Yes, Part-time | 15 | 19.74 |
| Yes, Full-time | 16 | 21.05 |
| Yes, not specified | 2 | 2.63 |
| Years in Program |  |  |
| Less than 1 | 12 | 15.79 |
| 1 | 37 | 48.68 |
| 2 | 20 | 26.32 |
| 3 | 4 | 5.26 |
| 4+ | 3 | 3.95 |
| Smoking Behavior |  |  |
| Non-smoker | 55 | 72.37 |
| Smoker | 19 | 25.00 |
| Cessate | 4 | 5.26 |
| Decrease | 4 | 5.26 |
| No change in amount | 11 | 14.47 |
| Missing | 2 | 2.63 |
| Total | 76 | 100.00 |

Appendix 2. Client interview characteristics by smoking status

|  | Non-smoker | | Smokers | |
| --- | --- | --- | --- | --- |
| Program Model | N | % | N | % |
| HFA | 7 | 13 | 4 | 21 |
| PAT | 15 | 27 | 3 | 16 |
| NFP | 28 | 51 | 6 | 32 |
| EHS | 5 | 9 | 6 | 32 |
| Site |  |  |  |  |
| Site 1 | 5 | 9 | 2 | 11 |
| Site 2 | 5 | 9 | 0 | 0 |
| Site 3 | 6 | 11 | 3 | 16 |
| Site 4 | 1 | 2 | 5 | 26 |
| Site 5 | 6 | 11 | 2 | 11 |
| Site 6 | 2 | 4 | 0 | 0 |
| Site 7 | 20 | 36 | 1 | 5 |
| Site 8 | 4 | 7 | 3 | 16 |
| Site 9 | 4 | 7 | 2 | 11 |
| Site 10 | 2 | 4 | 1 | 5 |
| Sex |  |  |  |  |
| Female | 51 | 93 | 18 | 95 |
| Male | 4 | 7 | 1 | 5 |
| Race |  |  |  |  |
| White | 28 | 51 | 15 | 79 |
| Black/African American | 20 | 36 | 3 | 16 |
| Other | 7 | 13 | 1 | 5 |
| Age |  |  |  |  |
| 18 and Under | 3 | 5 | 0 | 0 |
| 19-22 | 7 | 13 | 8 | 42 |
| 23+ | 45 | 82 | 11 | 58 |
| Employment |  |  |  |  |
| Unemployed | 31 | 56 | 10 | 53 |
| Employed | 24 | 44 | 9 | 47 |
| Marital Status |  |  |  |  |
| Single | 26 | 47 | 11 | 58 |
| Married/Partnered | 26 | 47 | 8 | 42 |
| Separated or Divorced | 3 | 5 | 0 | 0 |
| Education |  |  |  |  |
| High School or Less | 25 | 45 | 11 | 58 |
| Some College | 20 | 36 | 8 | 42 |
| College or Higher | 10 | 18 | 0 | 0 |
| Children |  |  |  |  |
| Pregnant | 4 | 7 | 0 | 0 |
| 1 | 33 | 60 | 10 | 53 |
| 2 | 10 | 18 | 5 | 26 |
| 3+ | 6 | 11 | 4 | 21 |
| Grandparent | 2 | 4 | 0 | 0 |
| Years in Program |  |  |  |  |
| <1 | 11 | 20 | 2 | 11 |
| 1-2 | 31 | 56 | 9 | 47 |
| 2+ | 13 | 24 | 8 | 42 |
| Total | 55 | 100 | 19 | 100 |

Appendix 3. Interview nodes and description

| Node | | Definition |
| --- | --- | --- |
| Smoking | | All content related to how home visiting addresses smoking with clients, as well as any content describing how clients feel about/react to that content. |
|  | Forms of support | Descriptions of the forms of support that home visitors/the program provide for people who need help with smoking. Could range from specific advice to informational pamphlets to referrals to cessation programs. |
|  | Harm reduction | Discussion of strategies that reduce and minimize the exposure of children to second and third hand smoke in and outside of the home. Make sure to code content related to the strategies themselves and the way participants and their families feel about and implement them. |
|  | Home visitor attitude | Content related to how the approach and attitude of the home visitor (or program in general) impacts how a client feels about or responds to discussions about smoking. |
|  | Important to discuss | Code responses to the question about how important it is for programs to address smoking here. |
|  | Individual readiness | Descriptions of how an individual's own commitment/readiness to reduce or quit smoking impacts their success. Also include statements from clients who smoke about their difficutly reducing or quitting that allude to their own commitment/readiness to change. |
|  | Learned new info | Client describes learning something about smoking, second-, or third-hand smoke. |
|  | Others | Content related to non-clients (family, friends, ets) smoking behaviors. |
|  | Stress relief | Any content where clients are describing the relationship between stress and smoking. |

| **Appendix 4. Supportive Quotes from Qualitative Interviews** |
| --- |
| Smoking Cessation |
| **“I don’t smoke anymore. …[My home visitor] helped me realize how really – how big of a risk it was to him. I mean, I knew – of course, I knew not to smoke around him and not to while I was pregnant. I knew that was harmful. But I didn’t realize that it can even get into – I guess like, into the walls and things like that, the room that he’s in. I didn’t realize that was important, also. So that helped.”** (3006; Black, 22)  “It's helped. **They actually help – well, kind of gave me suggestions of how to quit smoking with all my pregnancies. But I mainly did that by myself.** Because you’re supposed to gradually go and everything else. That’s what they're saying. They give you information – when you’re pregnant and you're smoking – about how to gradually go off and everything. …It was mainly information, but they also give out fliers for different programs, quitting programs in local areas that you're at.” (10003; White, 21)  “I was when I got pregnant, but as soon as I found out I was pregnant, I stopped.” (1005, White, 22)  “So as for smoking whenever I was pregnant, I quit immediately.” (4006, White, 37) |
| Smoking Reduction and Behavior Change |
| **Smokers:**  “I will admit I do smoke. I cut down a lot and, [home visitor], my home visitor, actually presented me with information quit packets. The 1-800 number for the PA helpline. What secondhand smoking can do to a child. why it’s important. …Obviously, don’t smoke inside your house. That’s part of child safety. I have a special smoking outfit that I wear whenever I smoke and I take it off before I interact with my son in type of way.” (8002; Asian, 23)  “I am a smoker – I was a smoker, but **I did cut back tremendously when I was pregnant for – per [home visitor’s] advice, and also, quit smoking in the house entirely and the car. We made that goal as well. …[W]e talked a lot about SIDS just because I was so inquisitive about it because I was a smoker. Anything negative that I wanted to learn and about the effects and what the best way was to quit, and hotlines I could call, and discuss with my doctor, and steps I should take to quit.** I just – **after I was able to smoke again I went to town.** …But we talked about that, too, and brought it back down. I mean that’s been a **constant struggle**.” (3007; White, 33)  I smoke. And she always tries to give me the number, get me to sign up for that smoke – well, those people that call you and help you quit. …She wants me to do it, and I wanna do it. But I just don't wanna do it right now. …Then even she gave me information about it and helped me talk to people to at least cut down a little bit… and I wish I would've kept up with it obviously. …I have a lot of stress now with everything going on. Not even just him, but – so then I just feel like I could go smoke a cigarette and calm down. …She gave me tips from her other clients – what they did to help themselves cut down. …When I was pregnant, it did help me cut down. …Like just taking a walk, thinking about other things, even chewing gum and stuff like that. Just anything basically to get my mind off of it.” (5008; White, 20)  “[B]efore I was pregnant, I did smoke, so it was a whole lot things that I didn’t know. So when I did get pregnant or once I had the baby, it gave me a sense of trying to keep smoke away from me or the baby as much as possible.” (7018; Black, 22)  “**We talked about third-hand smoke. Ah, yeah, she suggested the smoking jacket, which is the hoodie we keep outside for when we're smoking. Because I have like three, four feet of hair and it'll trap everything so – put the hoodie on when we go outside and have a cigarette and then take it off before we come in and wash our hands and everything. She's – yeah – I didn't even know third-hand smoke was a thing.** …She mentioned the amount of lead content in cigarette ash. So, now I make sure I sweep off the front porch – where we smoke on the front porch. I make sure I sweep the porch every day before the baby comes out. And with the back yard, people are allowed to smoke in the back yard as long as they use an ashtray. You can't just flick your ash in my yard anymore. Because he's – well, with him crawling around and putting his hands in his mouth. And that I didn't know about. She told me about that. So, that's another change.” (5002; White, 34)  “Definitely the second-hand smoke. Because she said like – I knew obviously about not smoking in the house with the baby. But even changing your clothes and stuff. I didn't know that. …Or wearing a sweater or something when you go out to smoke and then taking it off.” (5008; White, 20)  “I am a smoker and I will always be a smoker. But she knows that. …[Smoking and secondhand smoke] is awful. It truly is. I don’t like that I smoke, but it’s such an addictive habit and my stress reliever. I don’t know what I would quite do without it. And **I know that secondhand smoke is bad for my child which is why I smoke outside, and I wear a jacket and that jacket is the designated smoking jacket.** But it’s not a baby jacket, it’s not a let’s go eat in this jacket, it’s only for smoking. And I wash my hands and all that jazz.” (1001; White, 20)  “Well, originally we smoked around the kids. And now we try to go outside or in a different room if – well, as much as possible, depending on the weather and different situations.” (10003; White, 21)  “I really wasn’t aware that even – because I don’t like smoking around my son. So I try to, you know what I mean, put him in his room and put on some cartoons for him and I’ll go in my living room. You know what I mean? My little area. And smoke a cigarette. But it’s just, I didn’t know that second-hand smoke still can affect him for being in the back of the room where – you know what I mean? Or get in the back of the house or whatever the case may be. So I try to smoke outside and I try to limit it from smoking in front of him because my nurse would always tell me that that was very bad.” (3003; Black, 20)  “[W]en I first got pregnant, we were in a one-bedroom apartment that we just smoked all over all the time, with no regard for anything. If I – I mean, if I got ash on the floor, I'd vacuum it up, but maybe I wouldn't. Maybe I'd rub it in with my shoe, you know? Now since we got pregnant, we were – that week we got pregnant, we were in the apartment up until I was about eight months pregnant. And when we moved into the house that we're in now, we – we stopped smoking in the car right away. But then we stopped – we didn't smoke in this house ever. Like we – and that – he didn't wash his – neither of us washed our hands. Probably we'd smoke a cigarette and eat with the same hand. And now, nothing like that.” (5002; White, 34)  **Non-smokers**  “Another thing she said was if someone who was smoking or was a smoker who might have had the residue on maybe their clothing, she said to place a blanket or a burp rag over the person’s shoulder if they were going to be holding him for a long period of time, so. I think that’s something I hadn’t really thought of.” (3009; White, 30)  “I didn’t realize how, I mean, not the direct active breathing in secondhand smoke, but on the clothes, I didn’t realize how actually that was super dangerous, too. But that was nice to know about. …[O]ccasionally, I go to my dad’s house. …And one of their friends smokes and wanted to hold him. And I wouldn’t. I was like, no. Because they came to the house, so they couldn’t go change or anything. So I stated that I wasn’t real comfortable with that. Which, before, I probably wouldn’t have thought anything about it, honestly. …**I didn’t really expect to be around anybody that smoked, But with the information she gave me, I felt comfortable saying that I wasn’t comfortable with that person holding him**.” (5001; White, 26)  “We talked about it a lot – secondhand smoke. Because a lot of my friends and people would smoke, and she told me what to do. If they go outside and have a cigarette, make sure they take off their coat when they're holding your kid. Make sure they're not smoking around your kid. Stuff like that. But it was always brought up just in case. …It was helpful – very helpful. Because I knew that – okay. Well, like they need to wash their hands thoroughly. Because usually after seeing people go outside, after they come back and they hold the kid. So…I just made sure that all my friends wore a certain jacket outside to go have a cigarette if they were around my kid. And come back and take it off and wash their hands.” (5007; White, 26)  “[M]y dad smokes, So she did mention the effects that his smoking could have on my children and either smoke lingering in clothes to be transferred to my children, because they’re so small that, you know, toxic levels would just increase exponentially and things like that. …I make sure that if someone smokes and they’re gonna handle my children, that they have something like a smoking jacket.” (7020; Black, 22)  “I didn’t smoke and nobody around me smokes. But it was also just good to know what could happen and to stay away from people and places with smoke.” (7008; Black. 24)  “[W]e have talked about secondhand smoke, though, because my father smokes, but he goes outside, That’s still just that whole – when he comes in and you can still linger, and I try to put my boys in a bubble.” (2001; White, 31)  “[My husband]’s a smoker, but he’s set in his ways and he won’t quit. …[I learned] that [second-hand smoke] can cause asthma. It can, you know, lead to a lot of different things. But I don’t let my husband smoke in the house. I also don’t let him smoke around our son. We’re trying to prevent him – prevent all that.” (1005; White, 22) |
| Barriers to Change |
| **Stress:**  “I am actually bipolar, so the whole relieving stress is very helpful. Yes, I smoke, but I smoke outside the house. I always have, so that's not been a big issue with that. Yeah, they gave me the lecture I shouldn't have smoked while I was pregnant. …We hear about that and people are going to do what they're going to do.” (4002; White, 29)  “The nonsmoking thing, she tried to give me pamphlets, like papers for it, but I was like, I do want to stop, but I don’t want to stop because I feel that, **to be honest, a cigarette is like a relief to me. Like when I’m so stressed out I light a cigarette up, I smoke it and, poof, I’m relieved from that problem. I mean, that’s just how I feel. I don’t – you know, I don’t do nothing else except for cigarettes.**” (8007; Hispanic, 26)  “Sometimes having – I'd say that it calms us down in most situations, especially with our family and households. **The cigarette is like, hey, this is my getaway stress reliever compared to hey, I don't got this and I used to do it. I don't have nothing now. I can't go outside and have that couple minutes to calm down type of thing.**” (10003; White, 21)  “I don’t like that I smoke, but it’s such an addictive habit and my stress reliever. I don’t know what I would quite do without it.” (1001; White, 20)  “I wish I would've kept up with it [reduced smoking] obviously. …I have a lot of stress now with everything going on. Not even just him, but – so then I just feel like I could go smoke a cigarette and calm down.” (5008; White, 20)  **Individual Readiness**  **Smokers:**  “I still smoke. I understand all the things about it. It's not good and all that. But they still come around me sometimes whenever I'm smoking.” (4001; White, 26)  “She told me it was bad for the baby when I was pregnant and stuff. And it’s bad for a baby when it’s – for kids and babies after they’re born and stuff. …I’m one that’s hard to quit smoking. I'm like my mom. I’m hard headed.” (4004; White, 24)  “They gave me the quote unquote lecture – I shouldn’t smoke while I’m pregnant. We hear about that and **people are going to do what they’re going to do.” (4002; White, 29)**  “**I think some people just don’t wanna quit,** or they don’t have time to talk to somebody.” (5008; White, 20)  **Non-smokers:**  “With some people, I think it’s how they're going to be regardless of who says what to them.” (2002; White, 30)  “I think that would be a very hard issue for the teacher to bring up, kind of. I think if you are, you kind of, you're set in your way with that. So I don't know what they would do. I'm sure they could give them a flyer, but they probably just throw them away.” (2003; White, 27)  “**I think a lot of people are addicted to smoking. And if they're not – if they don't wanna quit, somebody telling them the risks and the harmfulness is not gonna change anything. …[The program] could address it, but as far as any changes occurring? I don't see that happening with the family unless, like I said, unless they are – I think the harmfulness of it should be addressed, but if the parents aren't willing to change, it's not gonna happen.**” (5004; White, 34)  “I mean, everyone is going to – if you're smoking, it’s not really going to make a difference. You kind of have to want to quit on your own to be able to do it. So, I mean, they could tell you to quit as much as they wanted to, but you kind of have to want to on your own to be able to do it.” (5006; White, 20)  “[The program] could give them advice as to how to stop. But it’s up to the person if they want to stop or not. Or they could direct them to a program and it’s up to them if they want to go to the program or not.” (7012; Black, 20)  “I believe they have the capability, but I think that when it comes to an addiction, like if you're a smoker, I think that you're kind of like stuck in your ways. You have to really want to change in order for the program to work, you could educate anybody on anything, but they're not going to change unless they feel like it’s a problem.” (7013; Black, 23)  “You can’t stop anybody from smoking.” (9004; White, 32)  **Home Visitor – Parent Relationship**  **Smokers:**  “Normally with those conversations, it's more of quitting. Like the goal is that for anybody that has kids is to quit smoking and to get in a different program to quit smoking. But we smoke in different rooms than the kids or go outside. **So we have been trying to avoid those subjects. I mean, some of us in the house is willing to go outside, and it's – there's been multiple conversations about smoking and secondhand smoke and that, so … They really don’t feel comfortable, because mainly it sounds like, ‘Hey, you need to quit.’ And not everybody's willing to quit.**” (10003; White, 21)  “Some parents don’t want to hear about it…they’re feeling like they’re being condemned as a bad parent because they smoke, but yet there’s other parents out there that drink and do other worse things. And smokers seem to be the ones getting condemned on.” (9005; White, 39)  “And originally when he was first born, I was supposed to have a separate set of clothes to go smoke in, and then come in and change my clothes and so he didn't have any kind of contact with any of that. And just the normal stuff like that. No smoking in the car, smoking outside, shouldn't smoke at all. …Well, I never did change my clothes. I mean, I'm not going to have a whole separate – I'm not going to change my clothes every time I go out to go smoke, and then change them when I come back in. But I do go outside to smoke. And when we smoke in the car, we have the windows down, and I make sure my cigarettes over by the window so I can see the smoke going out the window. …[The program should] maybe touch on [smoking], but not like you know – maybe touch on it here and there, but not like be too aggressive with it, if you can understand what I'm trying to say.” (9006; White, 32)  **Non-Smokers:**  “**People that smoke, I consider that an addiction. So I almost think it would be more helpful for them to link them to resources for smoking cessation rather than making them feel guilty for their second-hand smoke affecting their children. And I think that’s the affect it would have.**” (9003; White, 31) “I mean, [my husband’s] been smoking since he was 13 and he’s 34. So it was, kind of, when I told him that we have discussed it, he got irritated. But it could have been, you know, he was coming off having cigarettes. And that’s where her and I had discussed about his attitude towards everything. And I just – she’s not, you know, pushy about, oh, you have to stop because of [Son] and his lungs and this and that. But she just, well, just FYI, this is this and this happens here. And I like how that is.” (6001; White, 26) “She just gave us the rundown of what we kind of already knew about secondhand smoke being bad for the children and harmful. She just gave us the whole speech about it. She was very nice about it. She did not judge us. She spoke to us like people and not like clients, so to speak. It was more friendly. And she brought it up. She used her own personal experiences to talk about stuff with us. Very personable. I don't think she could have done it any better.” (10001; White, 30) |
